# Not Just for Urine: A Versatile Tool for Foreign Body Removal

**DOI:** 10.7759/cureus.11536

**Published:** 2020-11-17

**Authors:** Vir Singh, Vishalakshi Lakshmanan, Jennifer Setlik, Teerin Meckmongkol

**Affiliations:** 1 Emergency Medicine, University of Central Florida College of Medicine, Orlando, USA; 2 Emergency Medicine, Ocala Regional Medical Center, Ocala, USA; 3 Pediatrics, University of Central Florida College of Medicine, Orlando, USA; 4 Emergency Medicine, Nemours Children's Hospital, Orlando, USA; 5 Pediatric Surgery, Nemours Children's Hospital, Orlando, USA

**Keywords:** swallowed foreign body, hirschsprung disease, foreign body retrieval, foley catheter, pediatric abdominal pain, pediatric ileostomy

## Abstract

The swallowed or aspirated foreign body is a common pediatric emergency medicine complaint for which emergency providers must be familiar with the intricacies of management. Most swallowed foreign bodies will harmlessly pass through the GI tract, but children with GI tract abnormalities may have an increased risk of object impaction. There are few reported cases of foreign object ingestion in children with GI tract abnormalities, specifically ostomies. The Foley catheter is a versatile tool that is easily accessible in the ED setting. We present a novel case of foreign body ingestion in an infant with a colostomy secondary to Hirschsprung’s disease managed with Foley catheter retrieval through an ileostomy stoma.

A 17-month-old infant presented to the ED with a chief complaint of an episode of bloody vomiting. He had a two-day history of increased irritability and intolerance of feeds with emesis after every feed. The child’s medical history is pertinent for Hirschsprung’s disease, for which the patient had a pull-through procedure shortly after birth and a revision of the pull-through. On physical examination, the patient’s ostomy was found to contain brown-green liquid stool. A small ovular mass was visualized at the stoma during crying episodes. Supine posteroanterior radiograph of the abdomen showed an oval-shaped radiolucency consistent with a metallic ingested foreign body at the site of the stoma. The foreign object was removed using a Foley catheter and forceps and was found to be a penny. The patient was observed and discharged without complications later that day.

Treatment of a symptomatic ingested foreign object requires careful consideration of the type of object present and its location in the body. In this case report, we discussed the removal of an ingested coin in a symptomatic 17-month-old infant with a history of ileostomy secondary to Hirschsprung's disease using a Foley catheter. In children with ostomies, prompt imaging and non-surgical removal may be an option to manage retrieval of these objects if the patient is stable and symptoms are not severe.

## Introduction

The ingested foreign body is a common pediatric emergency medicine complaint for which emergency providers must be familiar with the intricacies of management. There are a variety of factors that determine whether management will involve expectant management, attempted bedside extraction, or endoscopic or operative retrieval of the foreign body. Patients with gastrointestinal anomalies, such as Hirschsprung's disease, introduce complexity into the standard management of such common complaints. The Foley catheter is a versatile tool that has been demonstrated to be useful in foreign body extraction from the esophagus among other locations. We present an interesting presentation of a swallowed foreign body in an infant with a diverting ileostomy secondary to Hirschsprung's disease, managed with Foley catheter retrieval.

## Case presentation

A 17-month-old infant presented to the ED with a chief complaint of an episode of bloody vomiting. The medical history is pertinent for Hirschsprung's disease, for which the patient has had multiple interventions. He had a pull-through shortly after birth and developed a rectal stricture. He had a revision of the pull-through after several months with the formation of a protective diverting ileostomy. 

The patient had been noted the day prior to arrival to have increased fussiness and intolerance of feeds at daycare. At home, he continued to be more irritable than usual, with long episodes of crying. The patient’s mother denied the patient bringing knees into his chest during episodes. She did not notice any periods of cessation of breathing and did not notice any cyanosis. She did feel that there was a ‘bulging’ underneath the ostomy stoma during episodes. The parent attempted to feed the child and the child vomited every time he attempted to eat. Vomit was yellow-colored, non-bilious, at this time non-bloody. He did tolerate some liquids. He had multiple wet diapers, though less than usual. Vomiting and poor feeding continued into the day of presentation, and the mother noted that the last episode of vomiting was ‘maroon’ or ‘rust’ colored, which concerned her for blood. 

The patient has no other pertinent medical history and takes no other medications. The review of systems was only positive for mucus-like discharge from the anus. This had been previously worked up two weeks prior to the anorectal examination under anesthesia, with the patient placed on amoxicillin/clavulanate for five days, which was completed.

On examination, the patient’s initial temperature was 98.1°F, heart rate was 158 beats per minute, respirations were 28 respirations per minute, and the oxygen saturation was 100% on room air. No blood pressure was recorded. The patient appeared to be irritable but consolable. Tympanic membranes were normal. Mucous membranes were dry. Tachycardia was noted. Lungs were clear to auscultation with no respiratory distress. The patient was observed to have an ostomy with brown-green liquid stool present. A small ovular mass was noted at the stoma during crying episodes. The abdomen was soft and non-tender. The penis and scrotum had no apparent pathology.

**Figure 1:**
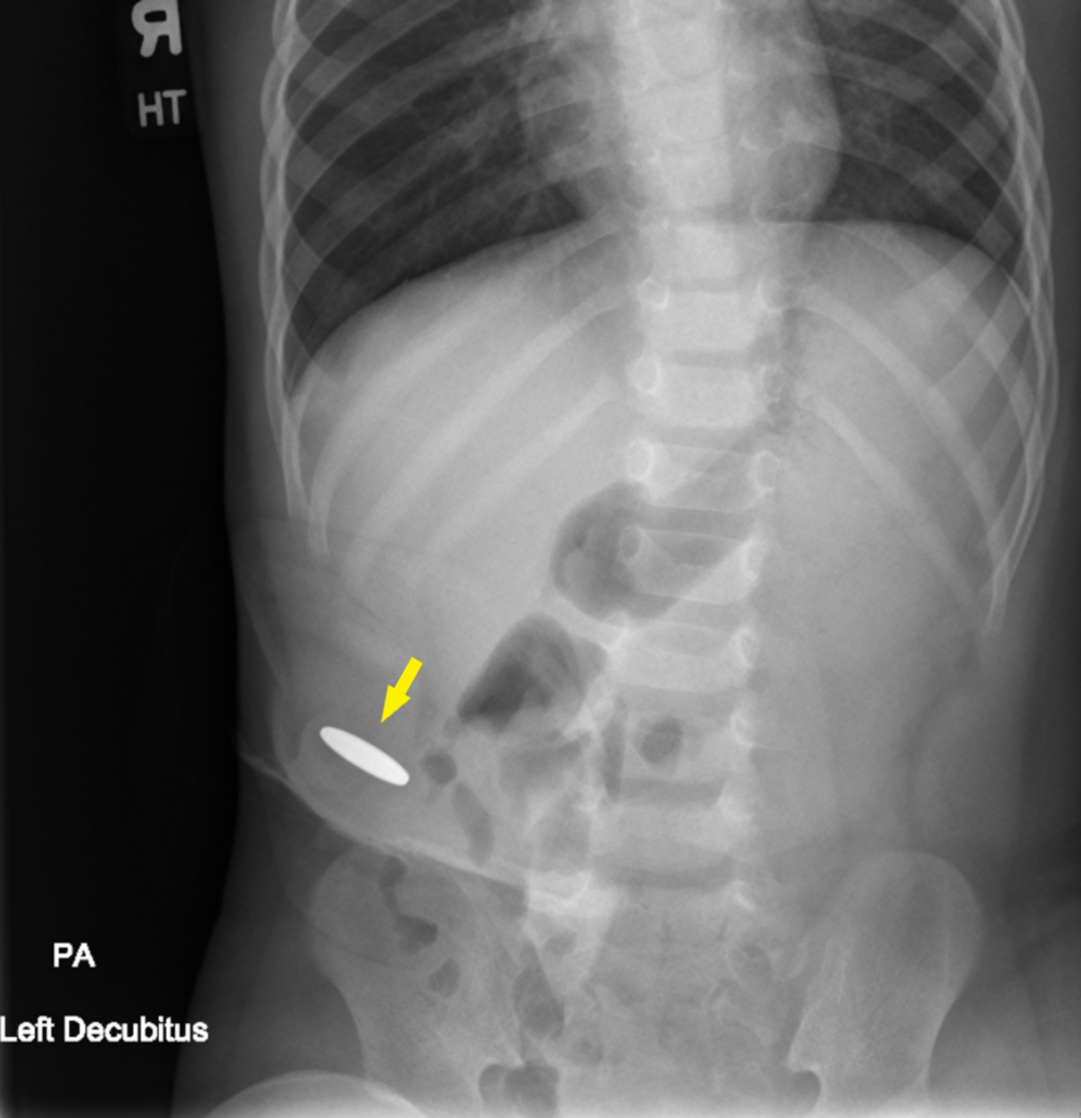
PA Abdominal Radiograph in Left Decubitus Position Demonstrating Oval Radiopaque Foreign Body PA: posteroanterior

The patient was assessed to be a child with a history of Hirschsprung's disease with diverting ileostomy presenting with increased crying, feed intolerance, and vomiting resulting in hematemesis, with tachycardia and a mass at the site of the stoma. Differential diagnoses included intussusception, volvulus, foreign body ingestion, colitis, Mallory-Weiss tear, and non-accidental trauma. The patient was placed on a cardiac monitor and intravenous access was obtained. Initial diagnostics ordered included complete blood count, comprehensive metabolic panel, urinalysis, and posteroanterior (PA) and lateral radiographs of the abdomen. A surgical consultation was placed. Initial treatment included a 20 mL/kg bolus of normal saline. The laboratory analysis demonstrated no anemia, no leukocytosis, no elevated transaminase, no elevated bilirubin. Potassium was elevated at 5.0 mmol/L (reference range 3.3-4.6 mmol/L). Blood urea nitrogen was elevated at 18 mg/dL (reference range 5-17 mg/dL). Creatinine was elevated at 0.4 mg/dL (reference range 0.10-0.36 mg/dL). PA radiograph of the abdomen in the left decubitus position (Figure 1) showed an oval-shaped radiolucency consistent with a metallic ingested foreign body at the site of the stoma. 

**Figure 2:**
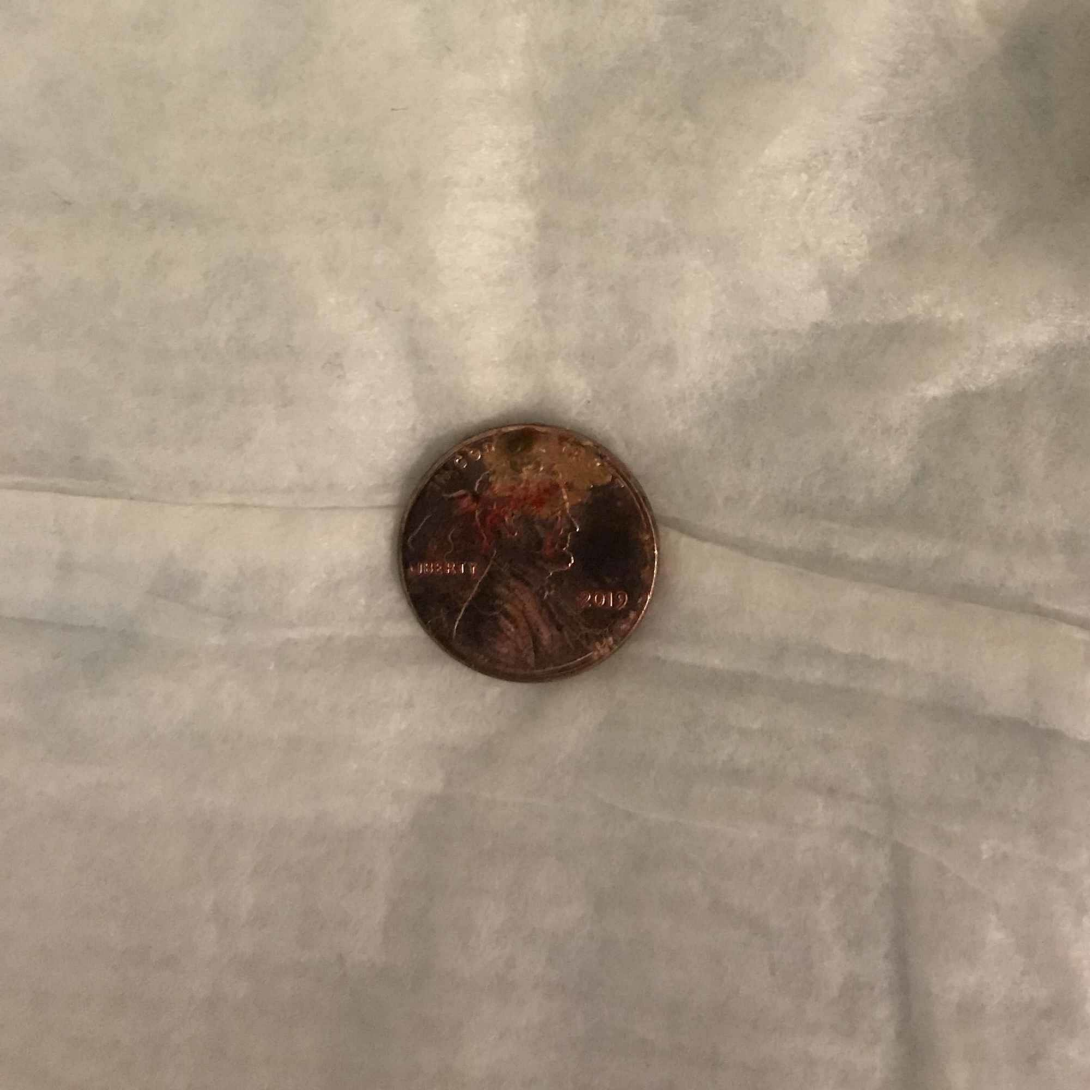
Ingested Penny Removed From Stoma via Foley Catheter Retrieval Technique

To remove the object, the pediatric surgeon utilized a Foley catheter foreign body retrieval method. A Foley catheter was inserted and guided blindly (without fluoroscopic or ultrasound assistance) past the foreign body, inflated, and retracted to bring the foreign body to the stoma opening. Forceps were then used to remove the foreign body through the stoma. The foreign body was found to be a penny (Figure 2). The patient was further observed in the ED and appeared well during his observation. He was discharged with follow-up to occur at the surgery clinic.

## Discussion

Accidental foreign body ingestion is a serious concern for children between the ages of 18 to 48 months, with coins being the most common foreign body ingested [1,2]. Over the course of nine years, there were 252,000 visits to the ED due to coin ingestion, with 20 fatalities in patients all under four years of age [3]. In children with normal anatomy, 80-90% of small objects that move past the pylorus will pass in the stool asymptomatically [4]. Objects that do not pass spontaneously are at risk for impaction in the GI tract, most commonly at the upper esophageal sphincter, an area of physiological narrowing [5]. The symptoms of foreign body ingestion in children are dependent on the number, type, and size of object ingested, its location in the body, and the length of ingestion. Symptoms can range in severity from drooling, refusal to eat, throat pain, and vomiting to respiratory distress and chest pain. Diagnosis of ingested radiopaque objects such as coins can be made with the use of anteroposterior and lateral radiographs in all patients.

Intervention may be necessary in symptomatic cases to prevent serious complications such as gastrointestinal ulceration, perforation, and death. Small objects trapped proximal to the upper esophageal sphincter can be extracted using forceps, while objects distal to the sphincter may require endoscopic removal depending on the child’s anatomy [6]. Laparoscopic removal may be necessary for removing foreign bodies that are impacted in the GI tract such as small animal bones, and those that may cause damage to the GI tract, such as needles, magnets, and button batteries.

Hirschsprung's disease, also known as congenital aganglionic megacolon, is an intestinal disorder resulting from the congenital absence of ganglion cells in the colon. The aganglionosis is attributed to the failure of neural crest cell migration to the gut during embryonic development. This disorder occurs in 1/5000 live births and is frequently diagnosed shortly after birth due to the infant’s failure to pass meconium in the first 48 hours of life [7]. Other symptoms at presentation can include emesis, abdominal distention, and intestinal obstruction due to the inadequate relaxation of the bowel wall. Hirschsprung's disease can be classified into short-segment disease and long-segment disease based on the length of the colon affected. A short-segment disease affecting only the rectum and sigmoid colon is more common and is present in up to 85% of patients with Hirschsprung's disease [8].

Currently, surgical intervention is the definitive treatment for Hirschsprung's disease. The surgery is usually done shortly after diagnosis, but timing depends on the presence of an identifiable transition zone and the prematurity of the infant. The pull-through procedure removes the diseased portion of the intestines and pulls the normally innervated intestines down to the anus. Surgical complications can include postoperative colitis, anastomotic leak, and/or strictures in 4-10% of patients [9]. Ostomies are often placed as a temporary measure in patients with complicated operative courses and those requiring multiple procedures. 

Although the swallowed foreign body is a common presentation in the general pediatric population, relatively less is known about the management and treatment of foreign object ingestion in patients with abnormal gastrointestinal anatomy, especially ostomies. One case report describes an 87-year-old woman who presented with bloody stool in her ostomy bag and no signs of obstruction [10]. She spontaneously passed a 1.3 cm diameter glass marble through her stoma a few days later. No reported cases of foreign object ingestion in pediatric patients with ostomies were found in our literature search. 

The Foley catheter is a versatile tool that has found multiple uses in addition to its primary use as an indwelling urinary catheter. With respect to foreign body retrieval, literature reviews have been written detailing the well-known use of Foley catheters to remove foreign bodies such as coins from the esophagus, with one such literature review of ten years of attempted coin extractions with a Foley catheter yielding a 96% success rate in 337 cases with no complications [11]. Nasal foreign body removal has been described in case reports and the literature with a Foley catheter [12], with more specialized proprietary Foley catheter-based technologies available. Case reports describe Foley catheter use for removal of rectal foreign bodies [13], and Fogarty catheter use in removing urethral foreign bodies [14]. 

In this case, it appears that an ingested coin passed spontaneously through the gastrointestinal tract, arriving at the ostomy site and causing persistent symptomatology as it was unable to be expelled from the ostomy without novel intervention with the Foley catheter.

## Conclusions

This case demonstrates the presenting symptoms, such as nausea, vomiting, fussiness, of a case of a foreign body lodged at an ostomy site of an infant. Additionally, it demonstrates that the practitioner in such a situation may be able to attempt non-surgical removal using a Foley catheter-aided extraction method. Were the patient unstable, or the foreign body unable to be brought through the ostomy or to have other concerning features (sharp edges, button battery, etc.), exploratory laparotomy would be indicated.
